# T-Cell Interferon Gamma Responses to SARS-CoV-2 Following Infection with/Without Vaccination in a Tanzanian Population

**DOI:** 10.2147/IDR.S532514

**Published:** 2025-12-04

**Authors:** Lilian Nkinda, Godfrey Barabona, Mark Ndubi, Chihiro Motozono, Emmanuel Nkuwi, Doreen Kamori, Frank Msafiri, Posian P Kunambi, Elisha Osati, Benson R Kidenya, Harrison Chuwa, Frank Eric Hassan, Juma Kisuse, Sayoki Mfinanga, Mbazi Senkoro, Takamasa Ueno, Eligius Francis Lyamuya, Emmanuel Balandya

**Affiliations:** 1 Campus College of Medicine, Muhimbili University of Health and Allied Sciences, Dar-es-Salaam, Tanzania; 2 Division of Infection and Immunity, Joint Research Centre for Retrovirus Infection, Kumamoto University, Kumamoto, Japan; 3 Department of Microbiology and Parasitology, University of Dodoma, Dodoma, Tanzania; 4 Management and Development for Health, Dar Es Salaam, Tanzania; 5 Department of Internal Medicine, Muhimbili National Hospital, Dar-es-Salaam, Tanzania; 6 Department of Biochemistry and Molecular Biology, Catholic University of Health and Allied Sciences- Bugando, Mwanza, Tanzania; 7 Aga Khan Hospital, Dar-es-Salaam, Tanzania; 8 National Institution for Medical Research, Muhimbili Centre, Dar Es Salaam, Tanzania

**Keywords:** SARS-CoV-2, ELISpot assay, high background, immune activation, Africa, Tanzania

## Abstract

**Background:**

T-cell responses are crucial in SARS-CoV-2 immune-control; however, limited data exist from African populations. We assessed interferon-gamma (IFN-γ) release by T cells among Tanzanian adults (18–70 years) previously infected with or without SARS-CoV-2 vaccination, using the ELISpot assay. We also characterized background plasma IFN-γ levels in this population.

**Methods:**

Peripheral blood mononuclear cells (PBMCs) from 143 individuals, sampled 1–12 months post SARS-CoV-2 exposure, were stimulated with overlapping peptides spanning the Spike and Nucleocapsid proteins. T-cell responses were measured by ELISpot assay, and plasma IFN-γ concentrations by ELISA. Associations with participant characteristics were analyzed using gamma linear and modified Poisson regression models (p < 0.05 considered significant).

**Results:**

We found high background T-cell IFN-γ release in 73.4% (105/143) of participants, leaving 38 (26.6%) with detectable responses above background; (38/38;100%) to Spike and (36/38;94.7%) to Nucleocapsid peptides. T-cell response magnitude did not differ by symptomatic/asymptomatic infection or vaccination status. However, each one-year increase in age was associated with a 1% decline in mean T-cell response (p = 0.029). Moreover, among participants with high background responses, 43/105;41% had elevated plasma IFN-γ, and 5/105; 4.8% showed cytokine storm-level concentrations. Alcohol consumption was significantly associated with elevated plasma IFN-γ (p = 0.041).

**Conclusion:**

Strong and possibly cross-reactive T-cell responses to SARS-CoV-2 were detected in Tanzanian individuals following infection with/without vaccination. Moreover, high plasma IFN-γ levels were detected, especially among participants who consumed alcohol. We recommend for modifications of the ELISpot T-cell assays to optimize the evaluation of pathogen-specific T-cell responses among African residents given the high background IFN-γ release.

## Introduction

Although neutralizing antibody titers are clearly correlated with protection against SARS-CoV-2 infection and severe illness,^1^,^2^ they gradually wane and have limited capacity in maintaining cross-neutralization potency against evolving variants of concern.^3^,^4^ According to Ahsanul. H. et al 2024, atleast 50% of Spike antibody responses wane off only four months post infection and vaccine receipt,^5^ whereas antibody against the Nucleocapsid are reported to wane even more rapidly reaching their half-life by 2.7 months.^6^ Despite this limitation, current data is largely restricted to neutralizing antibodies and data on other aspects of immune responses, including T-cell immunity, are still limited.^7^

Host T-cell defense against SARS-CoV-2 has been less intensively studied, and T-cell correlates of immune protection are yet to be established.^8^ One key challenge to proper characterization of the T-cell immunity is the variability in quantification assays^9^ as there is no WHO standardized guideline for quantifying SARS-CoV-2-specific T-cells, unlike for antibody response that is available^10^ although updates and harmonizations may still be needed. As a result, many laboratories rely on diverse in-house protocols, leading to varying results that complicate inter-laboratory and inter-population comparisons and in turn the evaluation of infection- and vaccine-induced immunity.^11^ Nonetheless, T-cell responses have been reported to persist for up to 17 years following infection with SARS-CoV-1 virus,^12^ and current data indicate T-cell responses generated against the ancestral SARS-CoV-2 virus from 2019 remain highly cross-reactive to a 2023 BA.2.86, Omicron variant with 60 amino acid changes compared to the original strain.^13^

Assessment of T-cell responses in most low resource settings is typically performed using the ELISpot assay.^14^ The ELISpot assay quantifies cells producing IFN-γ, a sole type II interferon primarily produced by primed CD4^+^ and CD8^+^ T-cells; these are primary subsets, critical for evaluating SARS-CoV-2-specific T cell immune responses following vaccination and/or infection.^11^ But also, a small cell population such as NK- and NK T cells could secrete IFN-γ and contribute to the spot formation. While the ELISpot assay is highly sensitive, cost-effective and requires minimal infrastructure, a 2012 credible EliSpot manual book highlighted that interpretation can become complex when either, the number of spots in peptide-containing wells is low, or spot counts in negative control wells are elevated, and particularly, when both of the aforementioned occur simultaneously.^15^ Initially, this ambiguity was attributed to methodological errors. However, as observed among the HIV infected individuals, the elevated background spot formation could potentially be a biological phenomenon, especially among relatively healthy African residents.^16^ While multiple studies have documented immune-activated phenotypes and heightened baseline inflammation in individuals of African descent,^17^ reports on elevated background immune activity affecting the interpretation of T-cell ELISpot data remain scarce. To the best of our search, only Liu et al *2018* have previously described this phenomenon, linking it to non-specific immune activation in a Kenyan population.^16^

Hence, this study reports T-cell responses to SARS-CoV-2 after infection with/without vaccination among adult individuals of African descent residing in Dar es Salaam, Tanzania. Moreover, we highlight challenges that could potentially be experienced when assessing T-cell responses among African residents using an ex-vivo interferon gamma release assay.

## Materials and Methods

### Study Site

We enrolled 150 participants from the Aga Khan Hospital, Mwananyamala and Temeke Regional Referral Hospitals and Muhimbili National Hospital in Dar-es Salaam Tanzania, from April to December 2022. Clinical data of hospitalized patients was extracted from patients’ charts. All laboratory analysis was conducted at Kumamoto University in Japan.

### Study Design and Study Population

This was a cross-sectional study.^18^ Adult participants above the age of 18 years, who were infected with SARS-CoV-2, with or without vaccination were recruited and categorized into asymptomatic infected-vaccinated (AIV), symptomatic infected-vaccinated (SIV) and symptomatic infected (SI). Vaccinated participants had received either Ad26.COV2-S (Janssen/Johnson&Johnson), BBIBP-CorV/Covilo (inactivated whole virus vaccine by Sinopharm) or BNT162b2 (BioNTech/Pfizer). All infected participants had PCR confirmation and were categorized as symptomatic if they presented with Corona Virus Disease-2019 (COVID-19) symptoms and were hospitalized. Asymptomatic individuals were categorized as so, after self-reporting not to have contracted COVID-19 but tested positive for nucleocapsid pooled peptide on the ELISpot assay. All infected-vaccinated individuals, regardless of symptoms, were recruited 1–12 months (median- 7 months) post-PCR diagnosis of SARS-CoV-2 infection and were vaccinated with the primary series vaccines 2–4 months (median- 2 months) prior or after confirmation of infection. Infected-only individuals were recruited for 1–8 months (median- 6 months) to post PCR test. In total, 150 participants, about 18–70 years were recruited into the study (30 SIV, 100 AIV and 20 SI). Moreover, symptomatic participants were categorized to have had severe COVID-19 if they had a respiratory rate greater than 30/min and oxygen saturation ≤93% at rest.^19^ Circulating variants at the time of data collection fell into the BA.2, BA.4 and BA.5 omicron sublineages^20^ similar to the neighboring country of Kenya.^21^

### Data and Sample Collection

Case report forms (CRFs) were used to collect baseline demographic data (age, sex, smoking habits, frequency of alcohol consumption), anthropometric measurements (weight, height), history of chronic illnesses such as diabetes, cardiovascular disorders, chronic renal disorders, chronic liver disease, any malignancy among others. We also collected information on COVID-19 infection and vaccine received (type of vaccine, and timing of vaccination and/or PCR confirmation of infection in relation to time of enrolment into the study) and clinical history of COVID-19 disease (if hospitalized and severity of disease). Participants’ blood was collected in Ethylenediamine tetra acetic acid (EDTA) coated tubes. Peripheral blood mononucleated cells (PBMCs) and plasma were subsequently isolated as described below.

### Isolation of PBMC and Plasma

PBMCs were collected as per the previously validated protocol between Tanzania and Sweden in 2008, with few modifications.^22^ Briefly, the EDTA blood was mixed 1:1 with Phosphate buffer sulphate (PBS) and carefully layered on the Ficoll histopaque and centrifuged for 30 minutes at 1020 x g, without brake or acceleration. Plasma was collected and stored at −80°C and for further laboratory analysis. PBMCs were carefully harvested and washed in pre-warmed R10 media which was RPMI 1640 (*Sigma, St. Louis, USA*) supplemented with 10% heat inactivated fetal calf serum (FCS). Thereafter, cells were resuspended in R10 media and stained using trypan blue and counted. Aliquots with at least 3 million live PBMCs per vial in freezing medium (10% dimethyl sulfoxide, 70% RPMI media, and 20% FCS) were gradually transferred to − 196°C liquid nitrogen from −80°C freezer within 24 hours.

## Measurement of T-Cell Immunity

### Peptides

For functional assays, PBMCs were stimulated using wild-type SARS-CoV-2 (D614G) antigens comprising 15-mer peptides pools (Miltenyi Biotec), overlapping by eleven amino acids of the full length of the Spike (S) and Nucleocapsid (N). The overlapping peptide (OLP) pools of S were composed of 315 peptides, while and N was composed of 102 peptides. All used peptides were made individually (SCRUM Inc) to ensure comprehensive screening as reported previously.^23^

### IFN Gamma ELISpot Assay

Interferon gamma (IFN-γ) ELISpot assay was performed on thawed PBMC samples as previously reported.^23^ Briefly, cryopreserved PBMCs were thawed, rested overnight and added at 200,000 cells per well in duplicates, and stimulated with 10 μL of SARS-CoV-2 peptide pools. PBMCs without peptide stimulation in duplicates were used as negative controls and the phyto-hematogluttin antigens were used as positive control. After 18 hours, IFN-γ release was detected by adding anti-IFN-γ biotinylated monoclonal antibody (*Mabtech*) for at least 2 hours, this was followed by addition of streptavidin alkaline phosphatase for 2 hours. Thereafter substrate was added. Finally, plates were scanned on Mabtech ELISpot Reader (v.4.0) and results were reported as spot-forming units (SFU) per million PBMCs. A positive result was calculated by subtracting SFU of stimulated wells from each respective negative control. Moreover, as an internal validation of the personnel technique, peptide quality and plate sterility, each plate was run with a sample of control participant who was a Tanzanian, but a resident in Japan for eight years, whose overall mean SFU of the negative control from the 14 ELISpot plates was 4.5. High background response was defined as high if the mean SFU in the duplicate negative control wells was ≥5 SFU/10^5^ PBMC.^16^

### IFN-γ ELISA Assay

Plasma human IFN-γ levels were assayed using Sandwich ELISA. Briefly, the anti-human IFN-γ antibody was coated on the 96 well plate. Subsequently, the plate was washed and blocked using phosphate buffered saline then washed and incubated with plasma samples, followed by washing and incubation with biotinylated antibody specific to human IFN-γ. Afterwards avidin horseradish peroxidase (HRP) conjugate was then added then substrate was added, Tetramethylbenzidine (TMB). The reaction was then stopped using sulphuric acid and optical densities (OD), were measured spectrophotometrically at a wavelength of 450 nm. The standard curve for calculating IFN-γ concentration was plotted using the four-parameter logistic model. Each assay plate was run with negative and positive control samples and standards in duplicates.

### Statistical Analysis

Gamma linear regression was used to analyze factors associated with T-cell responses and modified poison logistic regression was used to calculate factors associated with elevated plasma IFN-γ on the Statistical package for Social Sciences software version 27. The difference in medians of responders to Spike vs Nucleocapsid antigens was compared using Kruskal Wallis, test and post hoc analysis was used to determine intergroup differences. We used Spearman correlation rank to ascertain the relationship between T-cell IFN-γ release and plasma interferon gamma levels. P-values <5% were considered statistically significant on Prism 9 (GraphPad Software).

### Ethical Approval and Patient Consent Statement

Ethical approval to conduct this study was obtained from the National Institute for Medical Research (NIMR/HQ/R.8a/Vol.IX/4051) as well as MUHAS Institutional Review Board (IRB) (Ref.No.DA.282/289/01.C/). An informed consent in written format was obtained from each participant before being recruited into the study. Obtained data was stored in an encrypted file and participants were anonymized to ensure confidentiality. Importantly, the study was conducted in accordance with the Helsinki declaration.

## Results

### Baseline Characteristics of the Study Participants

A total of 150 participants were enrolled (Table 1). The participants were divided into three groups, as per their exposure, that is asymptomatically infected-vaccinated (n = 100) of whom majority [77% (77/100)] were below 50 years, more than half [65% (65/100)] were males, 37% (37/100) had other chronic diseases and a majority [71% (7/100)] were vaccinated with *Janssen* vaccine. The second group were symptomatically infected-vaccinated (n = 30), where similarly, majority [53% (16/30)] were below 50 years, 43% (13/30) were males, almost half [47% (14/30)] had severe disease, 54.8% (17/30) were suffering from other chronic diseases, and 85% (85/100) had received the *Janssen* vaccine (Ad26.COV2-S). Lastly, the symptomatic infected individuals (n = 20), 60% (12/18) were below 50 years, majority [60% (12/20)] were males, 44.4% (8/18) had the severe disease and 38.9% (7/18) had other chronic diseases.

**Table 1:** Baseline Characteristics of the Study Participants

Variable	n	Asymptomatic Infected Vaccinated (%)	Symptomatic Infected Vaccinated (%)	Symptomatic Infected (%)
Age (years)				
18–50	105	77 (77.0)	16 (53.3)	12 (60)
>50	45	23 (23.0)	14 (46.7)	8 (40)
Sex				
Male	90	65 (65.0)	13 (43.3)	12 (60)
Female	60	35 (35.0)	17 (56.7)	8 (40)
Smoking				
None	123	82 (82.8)	27 (93.1)	14 (82.4)
Current smoker	12	10 (10.1)	0 (0.0)	2 (11.8)
Past smoker	10	7 (71.)	2 (6.9)	1 (5.9)
Alcohol consumption				
None	79	50 (50.5)	18 (62.1)	11 (64.7)
Current drinker	31	23 (23.2)	4 (13.8)	4 (23.5)
Past drinker	35	26 (26.3)	7 (24.1)	2 (11.8)
BMI category				
Underweight (< 18.5)	9	9 (9.3)	0 (0.)	0 (0.0)
Normal (18.5–24.9)	77	55 (56.7)	11 (36.7)	11 (61.1)
Overweight (25.0–29.9)	31	21 (21.6)	6 (20.0)	4 (22.2)
Obese (≥ 30.0)	28	12 (12.4)	13 (43.3)	3 (16.7)
Severity of infection				
Yes	22		14 (46.7)	8 (44.4)
No	26		16 (53.3)	10 (55.6)
Chronic disease				
Yes	61	37 (37.8)	17 (54.8)	7 (38.9)
No	86	61 (62.2)	14 (45.2)	11 (61.1)
Type of vaccine				
Ad26.COV2-S vaccine	88	71 (71.0)	17 (85.0)	
BNT162b2 mRNA vaccine	14	11 (11.0)	3 (15.0)	
Sinopharm vaccine	18	18 (18.0)	0 (0.0)	

### T-Cell Responses Against SARS-CoV-2

**Figure 1:**
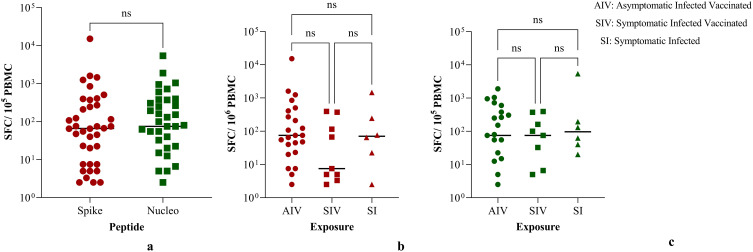
T-cell responses against the wild type SARS-CoV-2 variant. (**A**) T-cell responses against the spike peptides (red) and nucleocapsid peptides (green). (**B**)T-cell responses against the spike peptides in relation to the nature of exposure. (**C**) T-cell responses against the nucleocapsid peptides in relation to the nature of exposure. Comparisons of median were done using Kruskal Wallis test. P values are displayed as ns- not significant. SFU - is regarded as Spot Forming Units.

Seven (7) of the 150 participants were excluded from the analysis of SARS-CoV-2 memory T-cell responses because of low cell viability (<90%). Out of the remaining 143 participants, we observed high background IFN-γ SFU in unstimulated wells in 105/143 (73.4%) and only 38/143 participants (26.6%) had T-cell responses above background. In Figure 1A–C, we describe SARS-CoV-2-specific T-cell responses of these 38/143 (26.6%) participants. We hereby report that T-cell responses to Spike OLPs were detected in 100% (38/38) of the individuals, and (94.7% [36/38] individuals had T-cell immune responses against the nucleocapsid peptide. We observed no difference in the proportion of responders (p = 0.928) (Figure 1A). Moreover, there was no difference in the median T-cell responses in relation to the nature of exposure that is symptomatic or asymptomatic infected with/without vaccination for Spike (Figure 1B) or Nucleocapsid (Figure 1C) antigens.

### Univariate and Multivariate Analysis of Factors Associated with T- Cell Responses

Furthermore, we analysed factors that were independently associated with the strength of T-cell responses. On multivariate analysis, we found that age was significantly associated with the strength of T-cell responses whereby, for each one year increase in age, there was a 1% decrease in median T-cell responses (Table 2).

**Table 2:** Univariate and Multivariate Analysis of Factors Associated with T-Cell Responses

Variable	Univariate Analysis			Multivariate Analysis		
	cExp(β)	95% CI	p-value	aExp(β)	95% CI	p-value
Age in years	0.96	0.92–0.99	0.023	0.99	0.95–0.99	0.029
Male sex	0.38	0.14–1.04	0.060	0.63	0.23–1.70	0.361
Cigarette smoking	9.91	3.26–30.12	< 0.001	4.31	0.92–15.99	0.448
Alcohol consumption	5.00	1.95–12.81	< 0.001	2.48	0.89–6.90	0.082
Overweight and obese	1.26	0.45–3.50	0.664			
Severe infection	0.46	0.08–2.54	0.371			
Chronic disease	0.34	0.12–0.96	0.041			
Asymptomatic infected vaccinated*	0.94	0.24–3.72	0.932	1.83	0.55–6.10	0.324
Symptomatic infected vaccinated*	0.18	0.04–0.89	0.035	0.55	0.13–2.31	0.413

**Note**: *Reference category is symptomatic infected.

**Abbreviations**: cExp(β), crude Exponentiated beta coefficient; aExp(β), adjusted Exponentiated beta coefficient.

### High Backgrounds Interferon Gamma Release in the Study Population

We were unable to characterize SARS-Cov-2-specific T-cell responses in 105 out of 143 participants (73.4%) due to high background T-cell responses in the unstimulated wells. In detail, responses of 63.8% (67/105) of spike-stimulated wells, including 68% (49/98) of participants who had been vaccinated within the past 1–3 months, were masked – not above background. Additionally, approximately 36% (38/105) of nucleocapsid peptide responses also did not exceed background on the negative control wells and hence were excluded from the analysis of SARS-CoV-2-specific T-cell responses.

**Figure 2:**
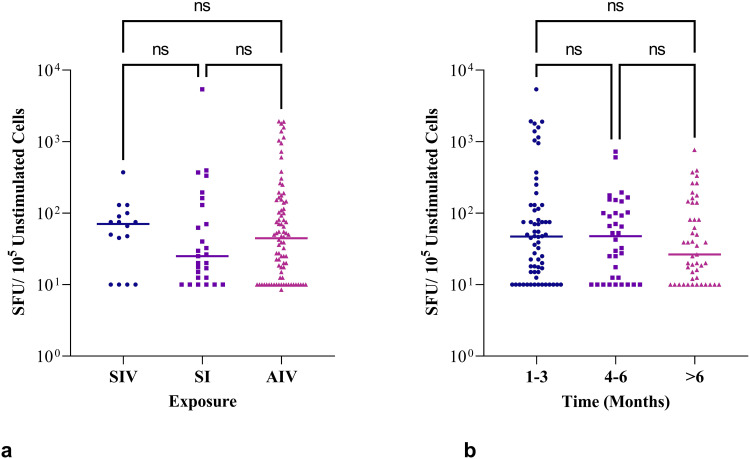
High background SFU in the unstimulated negative control wells. (**A**) Background T-cell IFN-γ release among participants by SARS-CoV-2 exposure (SIV = symptomatic infected-vaccinated, SI – symptomatic infected, AIV – asymptomatic infected-vaccinated). (**B**) Background T-cell IFN-γ release by duration since exposure to SARS-CoV-2 antigens via infection and/or vaccination.

**Abbreviations**: SFU, Spot Forming Units; Ns, not significant.

We therefore sought to further characterize the high background IFN-γ response in this study population. Figure 2A and B show the ELISpot readouts from the unstimulated negative control wells for the 105 participants, whose overall median baseline T-cell response exceeded 25 SFU/10^5^, which is higher than the ideal expected cut-off of 5 SFU/10^5^ or 50 SFU/10^6^ PBMC).^16^ The high backgrounds did not vary significantly based on nature of exposure or response duration, although lower backgrounds were observed in individuals who had longer duration (>6 months) since exposure to SARS-CoV-2 via infection or vaccination.

**Figure 3:**
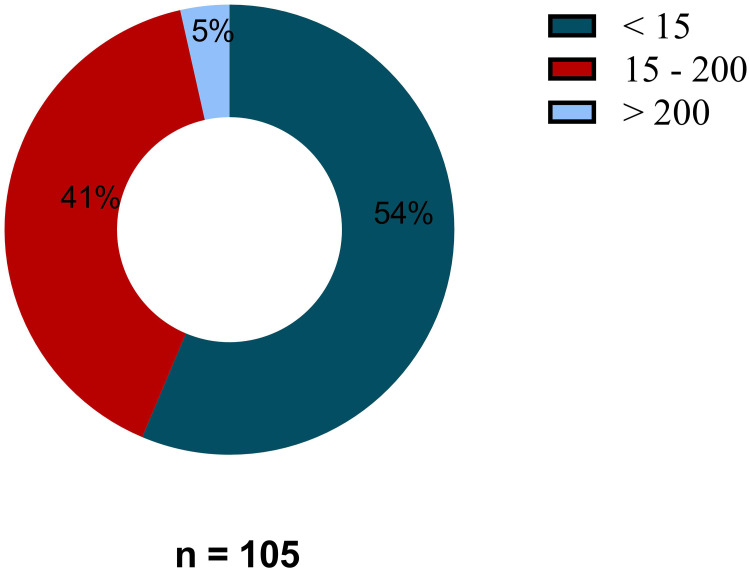
Elevated plasma interferon gamma. Red is the proportion of participants with elevated interferon gamma (15–200pg/mL) and blue the proportion of participants with normal interferon gamma (<15pg/mL), light blue are participants with cytokine storm (>200pg/mL).

To further assess the reason of the observed high baseline IFN-γ release, we quantified plasma interferon-gamma levels among the 105 participants via Elisa. As shown in Figure 3, nearly half of the participants (41%, 43/105) exhibited elevated plasma interferon-gamma levels (15 −200pg/mL), with 5% (5/105) of the participants had a frank cytokine storm (>200 pg/mL).

### Factors Associated with Elevated Plasma Interferon Gamma

On Table 3, we analysed factors associated with high plasma interferon gamma levels among the 105 participants whose SARS-CoV-2 T-cell responses did not exceed background. Because the outcome was common ie plasma interferon gamma was elevated in >20% of the participants, we used modified poison regression. Upon multivariate analysis, we observed that only alcohol consumption was significantly associated with elevated plasma interferon gamma levels (p = 0.041). Moreover, we did not find correlation between high background T-cell IFN-γ release and plasma interferon gamma levels (r = 0.1, p = 0.086).

**Table 3:** Factors Associated with Elevated Plasma Interferon Gamma Concentration

Variable	Category	Total	+IFN (%)	Univariable Analysis			Multivariable Analysis		
				cPR	95% CI	p-value	aPR	95% CI	p-value
Age	>50	18	8 (46.2)	1.16	0.60–2.25	0.654			
	18 – 50	87	32 (39.7)	Ref					
Sex	Male	73	28 (38.5)	0.80	0.46–1.39	0.436			
	Female	32	15 (47.8)	Ref					
Smoking	Smoker	7	1 (20.0)	0.46	0.80–2.75	0.141			
	None	98	42 (42.6)	Ref					
Alcohol	Drunker	41	23 (57.1)	1.83	1.07–3.15	0.027	1.53	1.02–2.29	0.041
	None	64	19 (31.1)	Ref					
BMI	Underweight	7	4 (45.5)	1.73	1.08–2.80	0.224			
	Overweight	28	12 (38.7)	0.90	0.54–1.50	0.673			
	Obese	11	5 (38.5)	0.89	0.51–1.54	0.677			
	Normal	59	22 (38.1)	Ref					
Severity of infection	Yes	20	9 (31.8)	0.72	0.34–1.54	0.400			
	No	25	11 (44.0)	Ref					
Chronic disease	Yes	18	11 (61.5)	1.53	1.06–2.22	0.024	1.02	0.67–2.17	0.933
	No	87	31 (36.1)	Ref					
Type of vaccine	Sinopharm	12	6 (82.4)	2.53	1.73–3.70	< 0.001	2.59	0.93–3.42	0.079
	mRNA	7	9 (71.4)	2.20	1.40–3.46	< 0.001	2.00	0.54–2.70	0.627
	Adenovirus	86	29 (34)	Ref					
Status	SI	15	7 (55.6)	1.20	0.75–1.91	0.445			
	SIV	22	10 (27.6)	0.60	0.32–1.12	0.106			
	AIV	68	31 (46.3)	Ref					

**Abbreviations**: cPR, crude Prevalence Ratio; aPR, adjusted Prevalence Ratio; Ref, Reference category.

## Discussion

We observed high background T-cell IFN-γ release in our study population. Nonetheless, robust T-cell responses to SARS-CoV-2 Spike and Nucleocapsid peptides were observed in a quarter of the study participants 1–12 months following infection with/without vaccination. The observed T-cell responses did not differ between those who were exposed to SARS-CoV-2 via infection alone compared to infection and vaccination, but the median response appeared to decrease by 1% for each one-year increase in age. Upon further characterization of the three-quarters of the participants with high background T-cell IFN-γ release, we observed that 41% also had elevated free plasma IFN-γ, and 4.8% had frank cytokine storm. Among the factors assessed, only alcohol consumption was independently associated with elevated plasma IFN-γ.

Despite the high background observed, we report robust infection and vaccine-induced T-cell responses against the wild-type SARS-CoV-2 virus among African inhabitants. Although the current circulating variant has undergone significant mutations compared to the wild-type strain, several reports have consistently shown that, even with the antigenic divergence between the ancestral strain and the more recent Omicron variant, T-cell responses have retained the ability to cross-react.^12^,^24–26^ Notably, a study by Geers et al 2024^27^ found that spike-specific T-cells cross-recognized over nine different SARS-CoV-2 variants this included: D614G, Delta, and omicron variants: BA.1, BA.2, BA.5, BQ.1.1, XBB.1.5, EG.5.1, and BA.2·86) both before and after vaccination.^27^ Similar observations were made in studies involving African populations, whereby T-cell responses against the endemic human coronaviruses (hCoVs) maintained robust responses (84%) against the recent SARS-CoV-2 in pre-pandemic samples.^28^ This suggests T-cell responses mounted against the WT variant may still exhibit some reactivity against the evolved recent variants. Secondly, it highlights the importance of T-cell mapping, along with sero-surveillance, to accurately identify immunity gaps at the population level.

Furthermore, we also observed high background T-cell IFN-γ release in most of the study participants, despite attempts to rest the PBMCs before the assay. Although no correlation was found between these high background responses and the elevated plasma IFN-γ levels, our findings suggest the presence of immune-activated PBMCs. Liu et al *2018*, previously showed high backgrounds in the ELISpot assay are significantly correlated with the presence of immune activated T-cell in the PBMCs.^16^ In their study, approximately 25% (n = 58) of ELISpot readouts from Kenyan participants had shown similar nonspecific high background responses in the negative control well.^16^ Persistent immune activation has been extensively documented among African residents, and it seem to be driven by environmental factors, such as continuous pathogen exposure, diet, and socioeconomic factors among others.^16^,^29^ For instance, past studies have demonstrated elevated expression of immune cell activation markers such as HLA-DR on CD4⁺ and CD8⁺ T-cells, as well as elevated CD38 expression on CD8⁺ T-cells, among HIV-negative Ethiopian immigrants compared to resident Israelis.^30^,^31^ Similar trends have been reported in Ugandans versus Italians^31^,^32^ and in Kenyans compared to Canadians, with the latter group displaying significantly higher frequencies of activated T-cell phenotypes and elevated IFN-γ production from both CD4⁺ and CD8⁺ T-cells.^31^,^33^ Consistent with these findings, our study extends these previous observations of heightened immune cell activation among African residents by reporting elevated IFN-γ as a key marker for T-cell functionality following immune cell activation.

While the ELISpot assay remains a cost-effective and widely accessible tool for assessing T-cell responses,^14^ chronic immune cell activation in this population necessitates caution when interpreting IFN-γ release assay results. Several studies have highlighted ambiguities at various stages of the immunological assay, from the pre-analytical, analytical, and post-analytical phases.^34^,^35^ Our findings further specify this immunological variability, particularly among African residents, potentially due to increased pathogen exposure, genetic predisposition, among other factors.^16^ Given that Africa is a critical region for the emergence and re-emergence of infectious diseases that could potentially have global implications, evaluating immune responses, especially T-cell immunity, is vital. These insights are fundamental for informing vaccine development and tailoring effective public health responses. As such, there is need for the ELISpot assays to be optimized to account for population-specific immunological backgrounds.

In our study, both current and past alcohol consumption were significantly associated with elevated levels of plasma interferon gamma. Previous studies have demonstrated a strong relationship between alcohol exposure and inflammatory biomarkers.^36–38^ On one hand, alcohol consumption is known to inhibit both type 1 and type 2 interferons by interfering with regulatory factors such as IRF-5 and IRF-7, as well as signal transducers in a 2010 study.^36^ On the other hand, consistent with the current study, there is substantial evidence showing increased interferon gamma production in mice exposed to alcohol compared to unexposed mice.^37^,^38^ Yue et al suggested that chronic alcohol exposure increases gut permeability, facilitating the translocation of gut microbiota, which in turn contributes to elevated inflammation^38^ consistent with the observations from our study. While moderate alcohol consumption was once considered safe, current recommendations from the World Health Organization state that there is no safe level of alcohol intake.^39^

We acknowledge several limitations in this study. Firstly, the use of frozen PBMCs may have contributed to the aberrant response, although we used a standard validated protocol for collection and storage^22^ informed by the manufacturer recommendations and previous publications, due to the unavailability of a global standardized guideline for evaluation. Also, we ensured that the cells had high viability and were rested overnight prior to analysis. Secondly, a limited sample size was used in assessing the T-cell responses as most samples were excluded from the analysis due to the high backgrounds. Further, due to low number of cells, we were unable to perform concurrent flow cytometric analysis that would have allowed us better resolution on the identity and phenotype of the IFN-γ producing cells. Nonetheless, our study fills an important gap by assessing SARS-CoV-2 T-cell responses in native Africans, a population with high genetic diversity and largely underrepresented in early vaccine evaluation studies. Understanding their immune responses is essential for guiding global vaccination strategies.

In conclusion, we observed robust T-cell responses against wild-type SARS-CoV-2 in an African population during the Omicron surge. These findings have public health implications, particularly in settings where frequent booster vaccinations with the emerging strains may not be feasible. Further, we confirm previous reports of high background T-cell IFN-γ release that calls for caution and possibly modification of the ELISpot assay while using it for evaluating T-cell responses, in populations with heightened baseline immune activity. Future research should consider profiling baseline pre-existing immune responses when developing immune assays or vaccines. Also, there is need to assess how the human Leukocyte antigen (HLA) polymorphism could influence antigen presentation and in turn shape T cell responses, especially in Africa where the HLA diversity is quite vast.

## Data Availability

Data used to draw this conclusion are available from the corresponding author on reasonable request.
